# Macrovascular Involvement in Systemic Sclerosis: Association Between Carotid Ultrasound Hemodynamics Parameters and Digital Ulcers

**DOI:** 10.3390/clinpract15080152

**Published:** 2025-08-18

**Authors:** Eugenio Capparelli, Francesco Lapia, Luca Clerici, Eleonora Zaccara, Giusy Cinzia Moltisanti, Francesca Capelli, Daniela Bompane, Laura Castelnovo, Antonio Tamburello, Maria Iacovantuono, Maria Sole Chimenti, Paola Maria Luigia Faggioli, Antonino Mazzone

**Affiliations:** 1 Rheumatology Unit, ASST Ovest Milanese, Legnano Hospital, Via Papa Giovanni Paolo II, Legnano, 20025 Milan, Italy; eleonora.zaccara@asst-ovestmi.it (E.Z.); daniela.bompane@asst-ovestmi.it (D.B.); laura.castelnovo@asst-ovestmi.it (L.C.); antonio.tamburello@asst-ovestmi.it (A.T.); paola.faggioli@asst-ovestmi.it (P.M.L.F.); 2 Rheumatology, Allergology and Clinical Immunology, Departement of Medicine of Systems, University of Rome Tor Vergata, 00133 Rome, Italy; mariaiacovantuono@gmail.com (M.I.); maria.sole.chimenti@uniroma2.it (M.S.C.); 3 Internal Medicine Unit, ASST Ovest Milanese, Legnano Hospital, Via Papa Giovanni Paolo II, Legnano, 20025 Milan, Italy; francesco.lapia@asst-ovestmi.it (F.L.); luca.clerici@asst-ovestmi.it (L.C.); giusycinzia.moltisanti@asst-ovestmi.it (G.C.M.); francesca.capelli@asst-ovestmi.it (F.C.); antonino.mazzone@asst-ovestmi.it (A.M.); 4 Department of Internal Medicine and Medical Therapeutics, University of Pavia, 27100 Pavia, Italy

**Keywords:** systemic sclerosis, digital ulcers, macrovascular impairment, cardiovascular risk, carotid hemodynamics

## Abstract

**Background:** Digital ulcers (DUs) are among the most debilitating vascular complications in SSc and are commonly attributed to microvascular damage. However, recent evidence suggests a potential involvement of macrovascular abnormalities, including subclinical atherosclerosis and altered hemodynamic parameters. **Objectives:** This study aimed to investigate the association between a history of DUs and macrovascular involvement in SSc patients through carotid and vertebral Doppler ultrasonography, with a focus on hemodynamic indices such as Peak Systolic Velocity (PSV), End-Diastolic Velocity (EDV), Resistive Index (RI), and Intima–Media Thickness (IMT). **Methods:** A cross-sectional study was conducted on 107 SSc patients. Clinical, serological, cardiovascular, and metabolic data were collected, and carotid–vertebral ultrasound was performed. Patients were stratified based on DU history. Statistical analyses assessed associations between DU status and carotid–vertebral US findings. **Results:** Patients with DUs showed a significantly higher PSV in both right (86.9 ± 67.9 vs. 64.2 ± 20.5 cm/s, *p* = 0.010) and left ICA (78.9 ± 29.6 vs. 63.4 ± 18.2 cm/s, *p* = 0.002). Right ICA RI vas elevated in the DU group (*p* = 0.021). PSV in the external carotid arteries was also bilaterally increased in DU patients (*p* < 0.005). DU-positive patients had a higher prevalence of left carotid plaques (*p* = 0.012) and right-sided ICA RI > 0.75 (*p* = 0.01). Logistic regression identified DU history as an independent predictor of PSV at ICA (β = 31.89, *p* = 0.043) and carotid plaque presence at any side (OR 14.34, *p* = 0.012). **Conclusions:** A history of digital ulcers in SSc patients is associated with altered carotid hemodynamics and an increased subclinical atherosclerotic burden. These findings suggest that DUs may reflect not only microvascular damage, but also macrovascular dysfunction, supporting the need for integrated vascular assessment in SSc clinical practice.

## 1. Introduction

Historically, vasculopathy in systemic sclerosis (SSc) was considered a microcirculatory disorder, however, accumulating evidence suggests that medium- and large-caliber arteries may also be implicated, revealing a complex scenario that extends beyond microcirculation [1,2]. Indeed, microvascular damage represents the hallmark of the disease, with endothelial dysfunction playing a pivotal role, even in the early stages [3]. The main clinical manifestations of SSc-related vasculopathy include Raynaud’s phenomenon (RP) and puffy hands, which are observed even in patients with a very early diagnosis of SSc (VEDOSS), while fingertip pitting scars, digital ulcers (DUs), telangiectasias, and pulmonary arterial hypertension (PAH) tend to appear in established forms of the disease [4,5].

Insightfully, DUs are among the most debilitating complications of SSc and have been widely acknowledged as markers of the severity of SSc-related vasculopathy. They may provoke significant pain, impaired hand function, increased risk of infection, and reduced quality of life in affected patients [6]. DUs are defined as the loss of epidermal continuity extending into the dermis, with different degrees of exposure of the underlying tissues, potentially evolving towards gangrene and digital loss [7].

In SSc, DU development has predominantly been attributed to microvascular injury, however, in the general population, the primary causes of digital ischemia include arterial abnormalities, extrinsic vascular compression, thromboembolic events, and atherosclerosis, with the latter predominantly resulting from plaque accumulation in large-caliber arteries [8,9,10,11].

In this context, recent research has shown that endothelial dysfunction is also present in the brachial arteries and correlates with microvascular damage at the nailfold level, suggesting a continuum of vascular injury spanning both micro- and macrovascular beds [12]. As shown in the Italian observational multicenter GIRRCS study, a slight increase in clinical and subclinical atherosclerosis was displayed by SSc patients compared to available controls. In addition, the authors demonstrated that both traditional cardiovascular risk factors and SSc-specific features, such as ischemic digital ulcers, played a synergistic role in the development of cardiovascular complications [13].

To detect pre-atherosclerotic changes, most studies have employed B-mode vascular ultrasound at carotid and peripheral artery beds [14]. These non-invasive techniques have proven effective in identifying early vascular abnormalities, such as Intima–Media Thickness (IMT) and arterial stiffness, which have been consistently observed at higher rates in SSc. Notably, these findings occur even in the absence of traditional cardiovascular risk factors and despite a relatively low incidence of clinically overt cardiovascular events in SSc patients [15].

However, Doppler ultrasound at both the carotid and peripheral artery levels could provide various hemodynamically significant indices other than IMT, including Peak Systolic Velocity (PSV), End-Diastolic Velocity (EDV), and Resistive Index (RI). These hemodynamic indices have already been validated as predictors of macrovascular dysfunction in other populations, such as Type 2 Diabetes Mellitus patients [16]. Briefly, PSV reflects the maximum blood flow velocity during systole and it is particularly useful in identifying areas of arterial narrowing [17]. EDV, on the other hand, represents blood flow velocity during diastole, and it is particularly sensitive to downstream vascular resistance [18]. RI, quantifying the resistance to blood flow within a vessel, is used to evaluate end-organ perfusion [19].

Lastly, given the conflicting data regarding which SSc-specific features best explain clinical or subclinical atherosclerosis and macrovascular impairment, our study investigates the association between DUs, widely recognized as a clinical surrogate of microvascular injury, and Doppler ultrasound indices of the carotid and vertebral arteries. Utilizing non-invasive Doppler hemodynamic measurements, including cIMT, PSV, EDV, and RI, our objective is to elucidate the emerging interplay between microvascular and macrovascular compartments. By employing widely accessible and routinely performed ultrasound imaging techniques, we further aim to support the integration of macrovascular ultrasound assessment into clinical practice for SSc patients.

## 2. Materials and Methods

### 2.1. Study Population and Sample Definition

This cross-sectional observational study involved a cohort of one-hundred and seven SSc patients attending the Scleroderma Unit of ASST Ovest Milanese, Legnano Hospital (Milan, Italy) comprising participants aged ≥18 years able to provide informed consent. Patients were selected based on their fulfillment of the 2013 ACR/EULAR for a definitive diagnosis of SSc [20]. Patients with severe heart failure, a history of congenital heart disease, current malignancies, or anti-neoplastic treatment and individuals who had undergone cardiac surgery, percutaneous coronary, carotid and vertebral intervention, pacemaker implant, or carotid–vertebral endarterectomy were excluded from the study. Severe cognitive impairment and pregnancy status served as further exclusion criteria.

Participants were recruited from September 2024 to May 2025, and the study was conducted in respect of the ethical guidelines proposed in the Declaration of Helsinki, with the approval of the Ethic Committee of Milan Area 3 (protocol number S00125/2023, 29 May 2023).

### 2.2. Data Collection

Patients’ demographic and anthropometric characteristics were extracted from medical records. Data collection included information on age, sex, weight, height, Body Mass Index, Body Surface Area, and previous or smoking status. Data concerning age at enrollment and SSc diagnosis were also gathered from clinical records, as well as disease duration.

Based on data from previous evaluations, all patients’ disease-specific characteristics were assessed, including the presence of RP, puffy hands, telangiectasias, prior and current history of DUs, fingertip pitting scars, sclerodactyly, skin sclerosis, calcinosis, microstomia and microcheilia, and musculoskeletal and upper and/or lower gastrointestinal involvements. Interstitial Lung disease (ILD), PAH, and cardiomyopathy were detected through chest High-Resolution Computed Tomography (HRCT), Right Heart Catheterization (RHC), and cardiac Magnetic Resonance Images (cMRI), respectively. The Modified Rodnan Skin Score (mRSS) was employed to assess skin sclerosis extension [21].

Moreover, for the main purpose of the study, the presence of prior and current DUs, defined as the presence of painful, well-demarcated areas of skin loss and/or ulceration occurring on the tips of the fingers or toes and resulting from ischemia due to severe microvascular damage, was recorded and selected as a hallmark of advanced microvascular involvement in our population for comparison analysis.

Serological classification based on positivity for anticentromere antibodies (ACA), anti-topoisomerase I (anti-Scl70) antibodies, and anti-RNA polymerase III antibodies (ARA) was collected from patient medical history. Based on the most recent assessment, Nailfold Videocapillaroscopy (NVC) patterns were classified according to the Cutolo criteria and categorized into early, active, and late patterns [22].

Current medication status with a potential influence on macro- and microvascular functionality was gathered, including anti-hypertensive treatment (such as calcium channel blockers (CCBs), angiotensin-converting enzyme inhibitors (ACEis), angiotensin receptor blockers (ARBs), beta-blockers, and diuretics), low-dose aspirin, intravenous Iloprost, endothelin receptor antagonists (ERAs), phosphodiesterase type 5 inhibitors (PDE5 is), and lipid-lowering treatment. Data on glucocorticoid usage and immunosuppressive treatments were also evaluated.

### 2.3. Cardiovascular and Atherosclerotic Risk Assessment

Data regarding comorbidities such as Type 2 Diabetes Mellitus, Systemic Arterial Hypertension, Arrhythmias, Hyperuricemia, and Dyslipidemia, with a known and established influence on the cardiovascular system, were taken into account, as well as a previous familial or personal history of cardio- and cerebrovascular events. Patients were clinically evaluated to obtain information regarding the presence of cardiopalmus, angina pectoris, and heart-related dyspnea.

All recruited participants underwent standardized measurements of hemodynamic parameters at rest, in a quiet environment, thirty minutes prior to the execution of the carotid–vertebral Doppler ultrasound. Specifically, systolic and diastolic blood pressure levels, as well as heart rate (HR), were recorded in duplicate, with each measurement taken five minutes apart using an automated oscillometric sphygmomanometer on the dominant arm after at least 10 minutes of supine rest [23]. Cardiovascular risk scores were calculated using the validated Framingham and ASCVD (Atherosclerotic Cardiovascular Disease) risk equations, incorporating clinical variables such as age, sex, blood pressure, lipid profile, diabetes status, and smoking habits, in accordance with ACC/AHA guidelines [24,25,26].

### 2.4. Biochemical and Metabolic Assessments

Venous blood samples were collected within the same month as the carotid and vertebral Doppler ultrasound examination. All laboratory analyses were performed in the institutional central laboratory, following standardized protocols. The metabolic and cardiovascular profile included measurements of total cholesterol, HDL cholesterol (HDL), LDL cholesterol (LDL), triglycerides, fasting glucose, high-sensitivity troponin T (hs-TnT), N-terminal pro–brain natriuretic peptide (NT-proBNP), uric acid, c-reactive protein (CRP), and hemoglobin (Hb). Units of measurement were as follows: cholesterol and triglycerides (mg/dL), glucose (mg/dL), troponin T (ng/L), NT-proBNP (pg/mL), uric acid (mg/dL), Hb (g/dL), and CRP (mg/L). Lipid parameters were assessed enzymatically, cardiac markers by electrochemiluminescence immunoassay, and Hb using an automated hematology analyzer.

Based on these parameters, the following metabolic indices were further calculated: (1) the triglyceride–glucose index (TyG index) was derived by taking the natural logarithm of the product of fasting triglyceride and fasting glucose levels divided by two [27]; (2) the LDL/HDL ratio was calculated by dividing the concentration of LDL cholesterol by that of HDL cholesterol [28]; (3) the triglyceride/HDL ratio (TG/HDL) was obtained by dividing serum triglycerides by HDL cholesterol [29]; (4) the Atherogenic Index of Plasma (AIP) was expressed as the base-10 logarithm of the ratio between serum triglycerides and HDL cholesterol [30]; and (5) the Homeostasis Model Assessment of Insulin Resistance (HOMA-IR) was calculated, where available, by multiplying fasting glucose (in mg/dL) by fasting insulin (in µU/mL) and dividing the result by 405 [31].

### 2.5. Ultrasound Examination

Ultrasound examination of the supra-aortic vessels was performed using a GE Vivid T8 ultrasound system equipped with a high-frequency linear transducer (8 MHz). All patients were examined in the supine position, with the neck slightly extended and rotated contralaterally to the side under evaluation to optimize image acquisition. For the common carotid artery (CCA), internal carotid artery (ICA), external carotid artery (ECA), and vertebral artery (VA), both transverse and longitudinal scans were performed using B-mode imaging, color Doppler, and pulsed-wave Doppler techniques.

Intima–Media Thickness (IMT) was measured in the longitudinal plane of the CCA, on the far wall, approximately 1 cm proximal to the carotid bifurcation [32]. The IMT value was calculated as the mean of three separate consecutive measurements. Atherosclerotic plaques were defined as focal structures that protrude into the arterial lumen by at least 0.5 mm or 50% of the surrounding IMT value, or that exhibit a thickness greater than 1.5 mm, measured from the intima–lumen interface to the media–adventitia interface [33]. For each vessel, Peak Systolic Velocity (PSV) and End-Diastolic Velocity (EDV) were recorded using pulsed-wave Doppler, maintaining an angle of insonation between 45° and 60°. In addition, Resistance Indices (RIs) were calculated for the CCA, ICA, and ECA to assess vascular resistance and aid in hemodynamic interpretation. The RI was calculated using the following formula: “RI = (PSV − EDV)/PSV”, and the validated cut-off of 0.75 was considered for the analysis [34].

In the presence of atherosclerotic plaques, the degree of stenosis was first assessed morphologically according to the criteria of the North American Symptomatic Carotid Endarterectomy Trial (NASCET) [35]. A complementary hemodynamic evaluation was also performed based on the classification proposed by Grant et al. and applied in cases of stenosis >50% or PSV >125 cm/s [36].

As most participants were undergoing monthly Iloprost infusion, US examination was performed two weeks after the last infusion to avoid any influence on SBP, DBP, and HR in examined velocities. Moreover, to avoid intra- and interobserver bias, the images were acquired and further evaluated two-fold by two experienced sonography examiners (F.L., seven years of experience, and L.C., five years of experience) who were blinded to patient data and characteristics.

### 2.6. Statistical Analysis

Continuous variables are expressed as mean ± standard deviation (SD) when normally distributed, or as median with interquartile range (IQR) in the case of non-normal distributions. Categorical variables are reported as absolute frequencies and percentages. Comparisons between continuous variables were performed using either the Student’s t-test or the Mann–Whitney U test, depending on the underlying data distribution. For categorical variables, the Chi-squared test or Fisher’s exact test was applied, as appropriate based on the sample size and expected frequencies. Linear regression analysis determined predictors for mean PSV at ICA and ECA. Additionally, a binary logistic regression model was developed to investigate potential risk factors associated with the presence of atherosclerotic plaques at any site. Covariates included in both analyses were selected a priori based on their well-established roles in plaque formation, as supported by evidence from the literature. The variables for the binary logistic regression comprised age, sex, BMI, LDL/HDL ratio, Framingham risk score, ASCVD risk score, and SBP, and as per the main purpose of the study, history of DUs was also incorporated. For the linear regression model, disease duration, AIP and SSc subtype according to the LeRoy classification were further included.

Statistical significance was defined as a *p*-value of ≤0.05 or when the 95% confidence interval excluded zero. The data analyses were conducted using IBM SPSS Statistics software, version 27 (IBM Corp., Armonk, NY, USA).

## 3. Results

### 3.1. Patient Clinical Features

A total of 107 patients were enrolled, with 76 (71.0%) having no history of DUs and 31 (29.0%) reporting past DUs. Female prevalence was significantly higher in the non-DUs group (96.1% vs. 71.0%, *p* < 0.001). Mean age at enrollment was similar between groups (62.3 ± 12.0 vs. 58.8 ± 12.7 years, *p* = 0.189), but patients with DUs were diagnosed earlier (43.4 ± 15.2 vs. 49.9 ± 13.2 years, *p* = 0.041). Disease duration, BMI, and BSA showed no significant differences (Table 1).

Regarding autoantibodies, anti-Scl-70 positivity was more frequent in the DUs group (35.5% vs. 7.9%, *p* = 0.001), whereas ACA and ARA antibodies were comparable. Skin involvement, measured by the mRSS, was greater in the DUs group (8.5 ± 8.4 vs. 2.1 ± 2.3, *p* < 0.001). According to the LeRoy classification, dcSSc was more common in the DUs group (38.7% vs. 1.3%, *p* = 0.001), while the limited and sine scleroderma subsets predominated in non-DU patients. NVC patterns also differed: early/active patterns were more frequent in non-DU patients (76.3% vs. 45.2%, *p* = 0.002), and late patterns prevailed among DU patients (54.8% vs. 23.7%, *p* = 0.002). Moreover, several clinical features correlated with a history of DUs, including puffy hands (*p* = 0.005), telangiectasias (*p* = 0.001), pitting scars (*p* < 0.001), sclerodactyly (*p* = 0.001), calcinosis (*p* = 0.003), friction rubs (*p* = 0.001), and microstomia (*p* < 0.001).

Upper gastrointestinal involvement was more prevalent in DU patients (80.6% vs. 56.6%, *p* = 0.019), while lower gastrointestinal symptoms, arthritis, renal crisis, cardiomyopathy, and PAH did not differ significantly. Lastly, ILD was notably more frequent in the DUs group (48.4% vs. 11.8%, *p* < 0.001).

Regarding therapies, antihypertensive medication usage and lipids and uric acid lowering therapies were comparable. The only differences emerged with the use of ERAs and sildenafil, as expected for the prevention of DUs (*p* < 0.001 for both) (Supplementary Table S1).

### 3.2. Cardiovascular Risk Assessment and Metabolic Indices

Patients with DUs more commonly reported angina pectoris (19.4% vs. 5.3%, *p* = 0.023) and hyperuricemia (16.1% vs. 1.3%, *p* = 0.003). However, no differences emerged for dyspnea, cardiopalmus, arrhythmias, hypertension, dyslipidemia, and T2 DM, as shown in Table 2.

**Figure 1:**
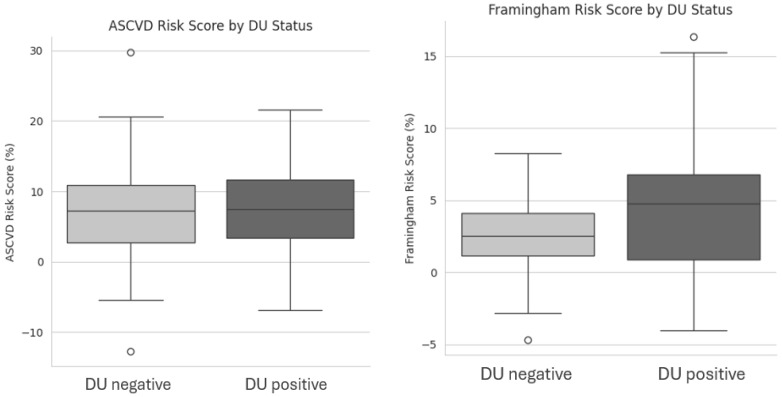
Box plot showing differences between DU negative and DU positive patients on ASCVD Risk Score and Framingham risk scores. Acronyms. DU = Digital Ulcers; ASCVD = Atherosclerotic Cardiovascular Disease.

Notably, metabolic parameters, including total cholesterol, HDL, LDL, triglycerides, fasting glucose, insulin, and calculated indices such as the TyG index, HOMA-IR, LDL/HDL ratio, TG/HDL ratio, and Atherogenic Index of Plasma (AIP), showed no significant differences between groups. Similarly, inflammatory and cardiac biomarkers (CRP, hs-TnT, NT-proBNP, and uric acid) were comparable. Blood pressure and heart rate, measured twice five minutes apart, did not differ significantly. Moreover, Framingham risk scores were higher in DU-positive patients (*p* = 0.048), indicating an increased cardiovascular risk, whereas ASCVD risk scores were similar, as shown in Figure 1.

### 3.3. Carotid Ultrasound Findings

**Figure 2:**
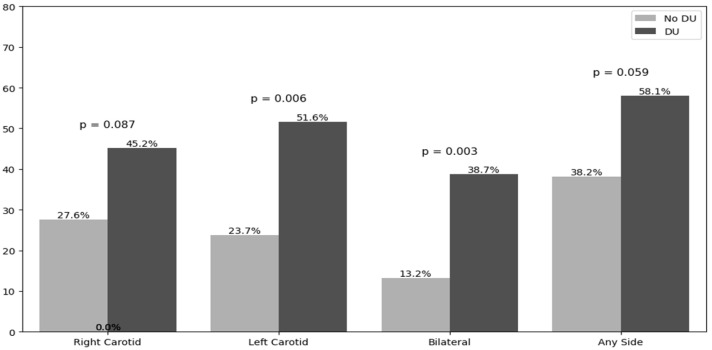
Atherosclerotic plaque distribution according to the side. Acronyms. DUs = Digital Ulcers.

Carotid ultrasound revealed a higher prevalence of atherosclerotic plaques in the left carotid artery in DU patients (51.6% vs. 26.3%, *p* = 0.012). Similarly, right carotid plaques were more frequent in those with DUs (45.2% vs. 31.6%), but without statistical significance. DU patients exhibited a greater prevalence of plaque bilateral localization (38.7% vs. 13.2%, *p* = 0.003), while the two groups did not differ when any side (left, right, or both) was considered, however, a trend toward significance was detected (58.1% vs. 38.2%, *p* = 0.059), as shown in Figure 2.

**Figure 3:**
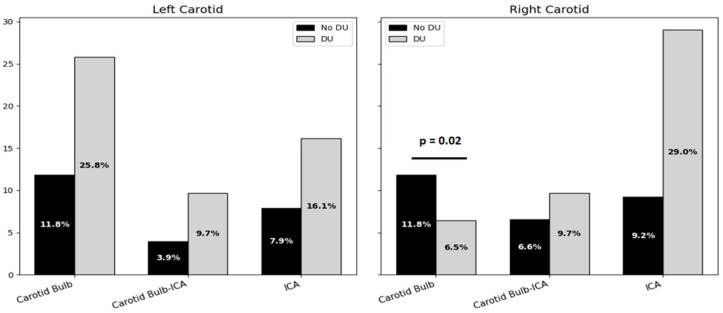
Bar chart on atherosclerotic plaque localization on both sides of carotid arteries. Acronyms. DUs = Digital Ulcers; ICA = Internal Carotid Artery.

On the left side, plaque prevalence in the carotid bulb was 11.8% in the non-DUs group and 25.8% in the DUs group, without reaching statistical significance. Plaques at the bulb–ICA transition were detected in 3.9% of non-DU and 9.7% of DU patients, while isolated ICA plaques were found in 7.9% and 16.1% of patients. In contrast, the right side revealed that carotid bulb plaques were significantly more frequent in patients with a history of DUs compared to those without (29.0% vs. 11.8%, *p* = 0.02). Plaques at the bulb–ICA site were seen in 9.7% of DU patients and 6.6% of non-DU patients (*p* = 0.69), and ICA plaques were seen in 29.0% vs. 9.2%, respectively (*p* = 0.07), showing a trend toward significance (Figure 3).

Moreover, the number of patients reporting a stenosis percentage at the plaque site comprising between 0 and 49% amounted to 18 out of 76 patients in the non-DUs group and 10 out of 31 in the DUs group (*p* = 0.4673), however, patients with a grade of stenosis more than 50% were 2/76 in the non-DUs group and 4/31 in the DUs group (*p* = 0.057), showing a trend toward significance in the latter group.

### 3.4. Doppler Hemodynamic Parameters

From a vascular standpoint, no significant differences were observed in EDV or cIMT measurements, as shown in Table 3. However, patients with DUs exhibited higher carotid blood flow velocities, still falling within the normal physiological range (PSV < 125 cm/s), with a significant bilateral increase in PSV of the ICA (right: 86.9 ± 67.9 vs. 64.2 ± 20.5 cm/s, *p* = 0.01; left: 78.9 ± 29.6 vs. 63.4 ± 18.2 cm/s, *p* = 0.001), as well as PSV in the ECA (right: 75.0 ± 24.2 vs. 87.7 ± 25.3 cm/s, *p* = 0.018; left: 71.7 ± 20.1 vs. 86.1 ± 24.1 cm/s, *p* = 0.002).

Moreover, significant increases in RI were noted in the right ICA in the DUs group compared to DU-negative controls (*p* = 0.021 and *p* = 0.013, respectively). Finally, the ICA/CCA PSV ratio on the right side was significantly elevated in DU patients (1.48 ± 1.21 vs. 1.16 ± 0.33; *p* = 0.043).

Furthermore, on the right side, DU patients more frequently had an elevated pulsatility index (PI > 1.2) and Resistive Index (RI > 0.75) in the ICA (35.5% vs. 13.5%, *p* = 0.01), along with more carotid stenosis (12.9% vs. 2.7%, *p* = 0.04). On the left side, an elevated PI and RI in the ICA were also more common in DU patients (35.5% vs. 8.1%, *p* < 0.001). No differences were found for cIMT > 0.9 mm, or elevated RI in the CCA or ECA, between the groups.

**Figure 4:**
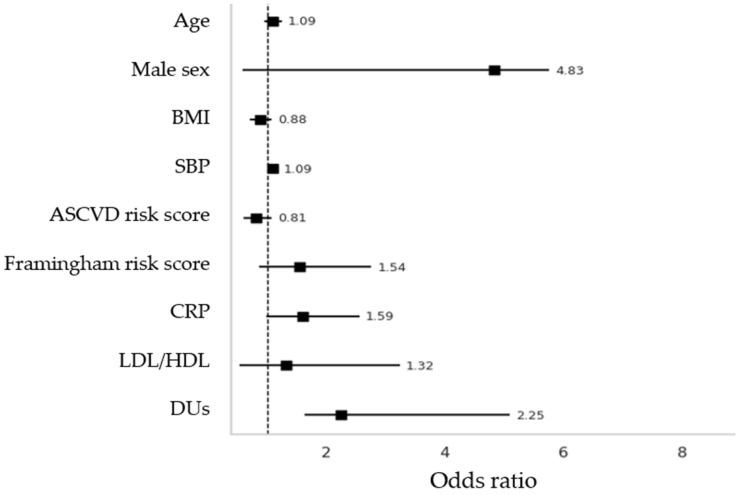
Binary logistic regression with adjusted OR with “plaques at any site” was considered as dependent variable. BMI = Body Mass Index; SBP = Systolic Blood Pressure; CRP = C-Reactive Protein; LDL = Low-density lipoprotein; HDL = High-density lipoprotein; ASCVD = Atherosclerotic Cardiovascular Disease.

Furthermore, on the right side, DU patients more frequently had an elevated Resistive Index (RI > 0.75) in the ICA (35.5% vs. 13.5%, *p* = 0.01), along with more carotid stenosis (12.9% vs. 2.7%, *p* = 0.04). On the left side, an elevated RI in the ICA was also more common in DU patients (35.5% vs. 8.1%, *p* < 0.001). No differences were found for CCA IMT > 0.9 mm or increased RI in the CCA or ECA between the groups, as shown in Figure 4.

### 3.5. Regression Analyses

Firstly, two multivariable linear regressions were performed to identify predictors of mean PSV at the ICA and ECA (Table 4).

For the ICA, a history of DUs was an independent predictor of a higher PSV (β = 31.89, *p* = 0.043). Traditional cardiovascular risk factors were not significant. In the ECA model, age predicted a lower PSV (β = −1.59, *p* = 0.022), while ASCVD risk score had a borderline positive association (β = 2.53, *p* = 0.055). DUs were not a significant determinant.

Secondly, in the binary logistic regression model employing plaques at any site as a dependent variable, it was revealed that both SBP and DUs were significantly associated with plaque presence (adjusted OR 1.09, *p* = 0.019; adjusted OR 2.25, *p* = 0.015, respectively) (Figure 4). Other included variables were age, sex, BMI, Framingham and ASCVD risk scores, and LDL/HDL ratio, which failed to prove associations.

## 4. Discussion

Our study provides evidence for the relationship between microvascular damage and macrovascular impairment in SSc from a peculiar clinical standpoint. Previous evidence has underscored that SSc patients exhibit increased cardiovascular mortality compared to healthy controls, and cardiac alterations can also be found in milder forms of the disease despite a lower prevalence of traditional cardiovascular risk factors across these populations [37,38]. In fact, cardiovascular mortality in SSc is estimated to be attributable to atherosclerotic events in up to 29% of cases, according to EUSTAR data, signifying a shift from SSc-specific causes (e.g., renal crisis and pulmonary hypertension) towards more generalized vascular complications [39,40,41].

Various studies have tried to define the SSc-related features which best contribute to macrovascular impairment and cardiovascular event prediction. For instance, a study by Caimmi et al., using ultrasound to analyze different medium-large vessels beds, such as carotid, upper, and lower limb arteries, revealed an association with Forced Vital Capacity, Diffusing Capacity of the lungs for Carbon Monoxide, limited cutaneous SSc, and calcinosis in defining macrovascular impairment [42]. In this context, particular interest has gained regarding the potential interconnection between microvascular changes, defined per the late NVC pattern or reduced capillary density, and altered endothelial function, detected as flow-mediated vasodilatation at the brachial arteries and arterial stiffness [12].

From a clinical perspective, the role of DUs in predicting macrovascular involvement in patients with SSc remains a subject of debate, with contrasting evidence reported in the literature. For instance, a large cohort study conducted on Japanese SSc patients failed to demonstrate a significant association between DUs and the development of atherosclerotic plaques. In contrast, data emerging from the GIRRCS study identified DUs as independent predictors of overt clinical atherosclerosis, although no significant link was found with subclinical atherosclerotic changes [13,43]. In view of this apparent dichotomy, we observed a higher prevalence of atherosclerotic plaques, particularly in the left carotid artery and bilaterally, among patients with a documented history of DUs.

In contrast to the Japanese study, despite its large sample size, our approach incorporated both hemodynamic assessments and cIMT evaluations, providing a more comprehensive analysis. The Japanese study primarily focused on the binary presence or absence of plaques, without integrating these additional parameters [43]. Furthermore, while the GIRRCS study emphasized the role of DUs in predicting clinically overt atherosclerosis, our findings suggest that DUs may also serve as a valuable predictor of subclinical carotid plaque formation, thereby reinforcing their significance as a marker of subtle macrovascular disease in SSc [13].

Moreover, although increased cIMT has long been recognized as a marker of cardiovascular morbidity and mortality, our findings suggest that alone, it may not suffice to stratify vascular risk in SSc patients [44]. Previous studies by Bartoli et al., Soltesz et al., and more recently Sedky Abdou et al. reported significantly increased cIMT in SSc patients compared to healthy controls, consistent with early arterial rearrangement toward increased stiffness. However, these changes do not always correlate with plaque presence or Doppler hemodynamic indices [45,46,47].

To clarify, in our analysis, only a few plaques determined significant hemodynamic alterations in the vascular beds under study, pointing to the presence of subclinical atheromatous process at this level. Moreover, this was elucidated by the comparable values of bilateral cIMT between the two groups, failing to reach the standardized cut-off of 0.9 mm indicative of atherosclerotic processes in the general population. This observation suggests that cIMT alone is not capable of defining plaque formation. This aligns with the previous findings of Frerix et al., who demonstrated discordance between plaque burden and cIMT in both SSc and systemic lupus erythematosus (SLE), suggesting that plaque formation may occur independently of intima–media thickening [48]. Moreover, Schiopu’s work noted increased expressions of serum proteins, including IL-2, IL-6, CRP, keratinocyte growth factor, intercellular adhesion molecule 1, endoglin, plasminogen activator inhibitor 1, and insulin-like growth factor binding protein 3, associated with carotid plaques in an SSc population, while myeloid progenitor inhibitory factor 1, serum amyloid A, thrombomodulin, N-terminal pro-brain natriuretic peptide (BNP), and Clara cell secretory protein 16 kD correlated with cIMT. Notably, these molecules are implicated in both fibrosis and vasculopathy processes, highlighting the presence of other intrinsic SSc-related mechanisms at play [49].

Supporting this hypothesis, the Doppler ultrasound findings in our cohort demonstrated an increased PSV in both the ICA and ECA among patients with DUs. Although these PSV values did not exceed clinically significant thresholds, their elevation, particularly when accompanied by a higher RI in the ICA, may reflect early alterations in arterial wall properties. Specifically, such changes suggest a reduction in arterial compliance in response to increased distal vascular resistance, even in the absence of critical stenosis.

Notably, the more prominent PSV alterations in the ICA and ECA, rather than in the CCA, are of particular interest. In this regard, vascular stiffness and endothelial dysfunction could preferentially affect arteries that are anatomically closer to the microvascular beds. The ICA and ECA serve as proximal conduits to microcirculation, and alterations in their hemodynamics may represent an indirect sign of impaired endothelial function. Thus, the closer the arterial segment is to the microvascular environment, the greater its susceptibility to resistance-related changes.

Definitively, a history of DUs might exert a proactive effect on determining these hemodynamic alterations occurring at the most distal branches of the carotid instead of CCA, which may reflect functional vessel stiffening and precede clinically overt atherosclerosis or ischemic events. These alterations have also been proven from a mechanistic point of view by Rollando D. et al., who demonstrated the presence of early-stage endothelial dysfunction at the brachial arteries in SSc, with parameters of altered FMD correlated to NVC changes [12]. In fact, endothelial cell injury induced by anti-endothelial antibodies, ischemia/reperfusion damage, and immune-mediated cytotoxicity represent the main causes of vascular injury, together with an impaired vascular repair mechanism, which determines defective vasculogenesis [50].

Collectively, these observations and ours reinforce the hypothesis that macrovascular impairment in SSc stems from a dual pathogenic origin: one is established by classical atherosclerosis, and the other is SSc-specific fibrotic vasculopathy. Remarkably, the observed increase in PSV, not paralleled by changes in EDV, points to a mechanism beyond simple luminal narrowing due to atherosclerosis, as increases in EDV are exclusively reported in proximity to atherosclerotic plaques [51].

Moreover, our research revealed that in DU patients, despite the presence of macrovascular alterations, no differences in classical cardiometabolic risk factors were found. Parameters such as the atherogenic index of plasma, TyG, HOMA-IR, and lipid ratios (TG/HDL and LDL/HDL) were similar between the groups, as were the rates of hypertension, diabetes, smoking, and dyslipidemia. DU patients had a higher cardiovascular risk as estimated by the Framingham score, but not by the ASCVD risk estimator. These discrepancies point to the inadequacy of traditional cardiovascular risk models in capturing the unique vascular pathology of SSc. Traditional models are primarily designed based on general population data, emphasizing risk factors such as lipid abnormalities, metabolic syndrome, and lifestyle-related contributors [52]. However, in SSc, the pathogenesis of vascular disease appears to follow a distinct trajectory, where immune-mediated endothelial dysfunction, vascular remodeling, and progressive fibrosis play central roles, often independently of typical metabolic derangements.

Another key consideration is the method of vascular assessment we employed. The use of carotid ultrasound and Doppler imaging provides a more sensitive evaluation of subclinical vascular pathology than broad estimators like Framingham or ASCVD risk scores do. In fact, Sanz Perez I et al. found that carotid ultrasound and coronary artery calcium (CAC) scoring were more effective in detecting subclinical atherosclerosis in SSc than conventional risk charts [53].

Our study presents several strengths. Firstly, the comprehensive evaluation of both microvascular (DUs and NVC) and macrovascular (carotid ultrasound, Doppler hemodynamics, and cIMT) parameters within the same cohort allowed for an integrated assessment of vascular pathology from a real-life clinical perspective. Second, the rigorous ultrasound methodology applied, with blinded dual-operator assessments, improved the reliability of imaging data and minimized operator bias.

Despite these strengths, several limitations should be acknowledged. For instance, the lack of standardized cut-off values for Doppler indices in SSc populations adds complexity to interpreting results and comparing findings across different studies. Future efforts should aim to elucidate common accepted thresholds in this cohort.

Although our primary objective was to explore whether clinical signs of microvascular dysfunction—specifically DUs—might serve as predictors of macrovascular alterations in SSc, our findings prompt the need for a more comprehensive assessment of endothelial function. In this regard, the inclusion of functional vascular tests such as FMD and arterial stiffness measurements would offer a more direct evaluation of endothelial health and vascular compliance.

Furthermore, it could be valuable to extend such vascular assessment to more distal arterial branches, such as the ophthalmic artery. Moreover, to elucidate the potential interconnection of macro- and microvasculopathy at the cerebral level, as recent evidence from optical coherence tomography angiography studies has shown a reduced retinal vascular density in patients with SSc and other autoimmune diseases, the employment of these advanced imaging modalities might provide further insight into this continuum [54,55,56].

The cross-sectional design of the study inherently restricts causal inferences regarding the temporal relationship between microvascular damage, macrovascular impairment, and cardiovascular events. Additionally, the single-time-point measurements may have missed dynamic changes in metabolic status or vascular health over time. Longitudinal follow-up would be necessary to clarify whether the observed vascular changes predict future cardiovascular morbidity and mortality in SSc. Finally, monocentric recruitment could limit the generalizability of these findings.

## 5. Conclusions

In conclusion, the data supports the concept that macrovascular disease in SSc arises from both atherosclerotic and fibrotic mechanisms. Traditional cardiovascular risk scores and metabolic parameters fail to account for this vascular burden, emphasizing the need for SSc-specific vascular assessment strategies. Incorporating DU status and non-invasive vascular imaging into routine clinical practice could allow for the earlier identification of patients at elevated risk, opening a window for timely preventive interventions and potentially improving cardiovascular outcomes in this high-risk population.

## Figures and Tables

**Table 1:** Clinical characteristics.

	Non-DUs n = 76	DUs n = 31	*p*-Value
Female, n/%	73/96.1	22/71	<0.001
Age at enrollment, mean ± SD	61.85 ± 12.4	58.8 ± 12.7	0.254
Age at diagnosis, mean ± SD	49.9 ± 13.2	43.4 ± 15.2	0.041
Disease Duration, mean ± SD	68.4 ± 335.6	15.0 ± 9	0.396
Body Mass Index, (Kg/m^2^), mean ± SD	23.1 ± 4.7	23.4 ± 4.2	0.813
Body Surface Area, (m^2^), mean ± SD	1.64 ± 0.18	1.69 ± 0.21	0.204
Anti-centromere, n/%	42/55.3	12/38.7	0.138
Anti-Scl70, n/%	6/7.9	11/35.5	0.001
Anti-RNA polimerase III, n/%	1/1.3	2/6.5	0.144
Current Smokers, n/%	17/22.4	8/25.8	0.703
mRSS at last follow up, mean ± SD	2.1 ± 2.3	8.5 ± 8.4	<0.001
LEROY CLASSIFICATION, n/%			
Sine scleroderma	26/34.2	2/6.5	0.001
Limited	49/64.5	17/54.8	0.001
Diffuse	1/1.3	12/38.7	0.001
NVC pattern, n/%			
Early/active	58/76.3	14/45.2	0.002
Late	18/23.7	17/54.8	0.002
Clinical Manifestations, n/%			
Puffy hands	60/78.9	16/51.6	0.005
Current Digital Ulcers	0	6/20	<0.001
Teleangctasias	30/39.5	23/74.2	0.001
Pitting scars	16/21.1	25/80.6	<0.001
Sclerodactily	33/43.4	26/83.9	0.001
Clacinosis	10/13.2	12/38.7	0.003
Friction Rubs	0	4/12.9	0.001
Arthritis	24/31.6	12/38.7	0.479
Upper GI Involvement	43/56.6	25/80.6	0.019
Lower GI Involvement	17/22.7	8/25.8	0.729
Microstomia	15/19.7	17/54.8	<0.001
Scleroderma Renal Crisis	1/1.3	1/3.2	0.508
Cardiomyopathy	0	1/3.2	0.116
Pulmonary Arterial Hypertension	1/1.3	1/3.2	0.508
Interstitial Lung Disease	9/11.8	15/48.4	<0.001

Acronyms. DUs = Digital Ulcers; mRSS = Modified Rodnan skin score; NVC = Nailfold Videocapillaroscopy; GI = Gastrointestinal.

**Table 2:** Atherosclerotic risk factors and metabolic indices.

	Non-DUs n = 76	DUs n = 31	*p*-Value
**Cardiovascular symptoms and related comorbidities**			
Angina pectoris, n/%	4/5.3	6/19.4	0.023
Dyspnoea, n/%	10/13.6	6/19.4	0.415
Cardiopalmus, n/%	7/9.2	4/12.9	0.568
Arrhytmias, n/%	19/25	13/41.9	0.083
Systemic Arterial Hypertension, n/%	22/28.9	13/41.9	0.194
Dyslipidemia, n/%	15/19.7	9/29	0.296
Type 2 Diabetes, n/%	2/2.6	2/6.5	0.345
Hyperuricemia, n/%	1/1.3	5/16.1	0.003
**Metabolic Assessment, mean ± SD**			
Total Cholesterol (mg/dL)	188.3 ± 41.6	187.4 ± 39.1	0.922
HDL-Cholesterol (mg/dL)	63.9 ± 16.5	60.5 ± 16.2	0.342
LDL-Cholesterol (mg/dL)	115.8 ± 31.5	113.3 ± 33.8	0.735
Tryglicerides (mg/dL)	96.9 ± 43.6	100.3 ± 48.1	0.748
Fasting Glucose (mg/dL)	91.6 ± 15.8	92.9 ± 29.1	0.773
Insulin	17.7 ± 26.8	10.9 ± 8.8	0.344
TyG index	8.3 ± 0.5	8.1 ± 1.4	0.361
c-LDL/c-HDL ratio	2.04 ± 1.81	1.94 ± 0.69	0.786
TG/c-HDL ratio	1.78 ± 1.54	1.73 ± 1.09	0.891
Atherogenic Index of Plasma	0.16 ± 0.27	0.14 ± 0.37	0.712
HOMA-IR Index	3.8 ± 8.1	0.8 ± 0.7	0.171
hs-TnT (ng/mL)	7.9 ± 5.9	10.1 ± 11.2	0.325
C-Reactive Protein (mg/L)	1.8 ± 2.2	2.7 ± 2.5	0.068
NT-proBNP, (pg/mL)	109.3 ± 106.6	139.9 ± 122.5	0.202
Uric Acid, (mg/dL)	4.4 ± 1.1	4.5 ± 1.3	0.678
1 st SBP (mmHg)	118.02 ± 16.4	121.8 ± 14.1	0.334
1 st DBP (mmHg)	78.8 ± 9.4	74.5 ± 13.0	0.094
1 st HR (bpm)	78.9 ± 11.9	75.2 ± 10.6	0.203
2 nd SBP (mmHg)	122.3 ± 17.1	123.1 ± 20.4	0.851
2 nd DBP (mmHg)	79.8 ± 11.3	76.2 ± 9.1	0.194
2 nd HR (bpm)	77.6 ± 9.8	75.8 ± 15.7	0.541
Familial CV events, n/%	19/25.3	9/29	0.694
Personal CV events, n/%	3/4.0	2/6.5	0.588
Framingham risk score, mean ± SD	2.9 ± 2.9	4.4 ± 4.4	0.048
ASCVD risk score, mean ± SD	6.7 ± 6.0	7.1 ± 6.6	0.787

Acronyms. DUs = Digital Ulcers; HDL = High-density lipoprotein; LDL = Low-density lipoprotein; TyG = Triglycerides–fasting glucose index; TG = Tryglicerides; HOMA-IR = Homeostasis Model Assessment- Insulin resistance; NT-proBNP = N-terminal-pro-Brain Natriuretic peptide; SBP = Systolic Blood Pressure; DBP = Diastolic Blood Pressure; HR = Heart Rate; CV = Cardiovascular; ASCVD = Atherosclerotic Cardiovascular Disease, hs-TnT = high-sensitive Troponin T.

**Table 3:** Doppler ultrasonographic hemodynamic parameters at carotid and vertebral levels.

	Right Scanning			Left Scanning		
Carotid–Vertebral US measurements	Non-DUs	DUs	*p*-value	Non-DUs	DUs	*p*-value
cIMT, mean ± SD	1.17 ± 3.03	0.82 ± 0.19	0.991	0.79 ± 0.19	0.85 ± 0.14	0.170
Common Carotid Arteries, mean ± SD						
Peak Systolic Velocity	56.7 ± 18.8	62.5 ± 16.9	0.172	58.7 ± 20.3	64.6 ± 21.0	0.205
End-Diastolic Velocity	15.9 ± 6.2	16.1 ± 7.1	0.973	17.1 ± 7.79	18.4 ± 7.9	0.482
Resistive Index	0.72 ± 0.06	0.74 ± 0.08	0.070	0.71 ± 0.08	0.72 ± 0.06	0.887
Internal Carotid Artery, mean ± SD						
Peak Systolic Velocity	64.2 ± 20.5	86.9 ± 67.9	**0.010**	63.4 ± 18.2	78.9 ± 29.6	**0.002**
End-Diastolic Velocity	21.2 ± 8.0	22.4 ± 10.4	0.544	23.2 ± 8.5	28.2 ± 23.7	0.129
Resistive Index	0.67 ± 0.07	0.71 ± 0.09	**0.021**	0.63 ± 0.07	0.61 ± 0.49	0.676
External Carotid Artery, mean ± SD						
Peak Systolic Velocity	75.0 ± 24.2	87.7 ± 25.3	**0.002**	71.7 ± 20.1	86.1 ± 24.1	**0.003**
End-Diastolic Velocity	16.5 ± 7.6	19.8 ± 12.8	0.113	14.9 ± 6.2	18.6 ± 8.3	0.143
Resistive Index	0.77 ± 0.08	0.68 ± 0.65	0.654	0.79 ± 0.06	0.79 ± 0.07	0.808
Vertebral Artery, mean ± SD						
Peak Systolic Velocity	38.1 ± 11.9	42.6 ± 12.1	0.218	39.5 ± 14.1	43.9 ± 13.9	0.277
End-Diastolic Velocity	11.6 ± 4.9	12.9 ± 5.5	0.230	13.2 ± 8.4	13.6 ± 6.4	0.394
Resistive Index	0.65 ± 0.36	0.71 ± 0.08	0.627	0.64 ± 0.37	0.70 ± 0.09	0.339
Carotid Stenosis percentage, mean ± SD	25.7 ± 14.3	35.6 ± 14.9	0.133	25.0 ± 011.1	27.8 ± 15.7	0.547
PSV ICA/CCA, mean ± SD	1.16 ± 0.33	1.48 ± 1.21	**0.043**	1.13 ± 0.31	1.25 ± 0.47	0.171

Acronym. DUs = Digital Ulcers; cIMT = carotid Intima–Media Thickness; ICA = Internal Carotid Artery, CCA = Common Carotid Artery. The bold font highlights the statistic significant *p*-values.

**Table 4:** Linear regression model predicting mean PSV at ICA and ECA as dependent variables (cm/sec).

	Mean PSV at ICA (Dependent Variable)			Mean PSV at ECA (Dependent Variable)		
	β-Coeff.	β-Stand. (95% CI)	*p*	β-Coeff.	β-Stand. (95% CI)	*p*
Constant	101.020	−54.71 to 256.75	0.197	157.78	29.82 to 285.75	0.017
Age	−1.14	−0.37 (−2.79 to 0.51)	0.169	**−1.59**	**0.70 (−2.95 to −0.24)**	**0.022**
Sex (Female)	10.89	0.11 (−31.46 to 53.25)	0.606	1.33	0.02 (−33.48 to 36.13)	0.939
BMI (Kg/m^2^)	−0.79	−0.09 (−3.51 to 1.92)	0.559	−0.25	−0.04 (−2.48 to 1.98)	0.821
Disease Duration	0.77	0.16 (−0.55 to 2.08)	0.246	0.11	0.03 (−0.97 to 1.19)	0.833
LeRoy SSc subtype (diffuse)	4.18	0.08 (−16.43 to 24.8)	0.684	−5.38	−0.13 (−22.32 to 11.55)	0.524
SBP (mmHg)	0.23	0.11 (−0.44 to 0.90)	0.491	−0.012	−0.01(−0.56 to 0.54)	0.965
CRP (mg/L)	−2.86	−1.22 (−11.23 to 5.52)	0.494	4.80	0.28 (−2.08 to 11.69)	0.166
AIP ratio	13.37	0.09 (−32.87 to 59.55)	0.563	1.81	0.02 (−36.16 to 39.79)	0.924
LDL/HDL ratio	0.67	0.02 (−14.22 to 15.56)	0.928	1.46	0.05 (−10.78 to 13.69)	0.811
Digital Ulcers	**3** **1** **.** **89**	**0.44 (1.03 to 62.76)**	**0.0** **43**	8.35	0.16 (−17.01 to 33.72)	0.509
Framingham Risk Score	−3.63	−0.33 (−9.23 to 2.006)	0.2	−1.78	−0.22 (−6.41 to 2.85)	0.442
ASCVD Risk Score	1.15	0.28 (−1.67 to 4.62)	0.348	2.53	0.65 (−0.06 to 5.11)	0.055

PSV = Peak Systolic Velocity; ICA = Internal Carotid Artery; ECA = External Carotid Artery; β-Coeff. = Beta-coefficient; β-Stand. = Beta-standardized coefficient; CI = Confidence interval; *p* = *p*-value; BMI = Body Mass Index; CRP = C-Reactive Protein; AIP = Atherogenic Index of Plasma; LDL = Low-density lipoprotein; HDL = High-density lipoprotein; ASCVD = Atherosclerotic Cardiovascular Disease. The bold font highlights the statistic significant associations.

## Data Availability

The data supporting this study’s findings are available from the corresponding author upon reasonable request. The data is not publicly available due to ethical restrictions.

## Supplementary Materials

The following supporting information can be downloaded at: https://www.mdpi.com/article/10.3390/clinpract15080152/s1, Table S1: Medication Usage.

## Abbreviations

The following abbreviations are used in this manuscript:

95%CI	95% Confidence Interval
ACA	Anti-Centromere Antibodies
ACC/AHA	American College of Cardiology/American Heart Association
ACEis	Angiotensin-Converting Enzyme Inhibitors
ACR/EULAR	American College of Rheumatology/European League Against Rheumatism
AIP	Atherogenic Index of Plasma
ARA	Anti-RNA Polimerase III Antibodies
ARBs	Angiotensin II Receptor Blockers
ASCVD	Atherosclerotic Cardiovascular Disease
ASST	Azienda Socio-Sanitaria Territoriale
β-coeff.	Beta Coefficient
β-stand	Standardized Beta Coefficient
BMI	Body Mass Index
BSA	Body Surface Area
CCA	Common Carotid Artery
CCBs	Calcium Channel Blockers
cIMT	Carotid Intima–Media Thickness
cMRI	Cardiac Magnetic Resonance Imaging
CRP	C-Reactive Protein
DBP	Diastolic Blood Pressure
DUs	Digital Ulcers
ECA	External Carotid Artery
EDV	End-Diastolic Velocity
ERAs	Endothelin Receptor Antagonists
GIRRCS	Gruppo Italiano per la Ricerca e la Ricerca Clinica sulla Sclerodermia
HDL	High-Density Lipoprotein
HOMA-IR	Homeostatic Model Assessment of Insulin Resistance
HR	Heart Rate
HRCT	High-Resolution Computed Tomography
hs-TnT	High-Sensitivity Troponin T
ICA	Internal Carotid Artery
ILD	Interstitial Lung Disease
IMT	Intima–Media Thickness
IQR	Interquartile Range
LDL	Low-Density Lipoprotein
mRSS	Modified Rodnan Skin Score
NASCET	North American Symptomatic Carotid Endarterectomy Trial
NT-proBNP	N-terminal pro Brain Natriuretic Peptide
NVC	Nailfold Videocapillaroscopy
PAH	Pulmonary Arterial Hypertension
PDE5 i	Phosphodiesterase Type 5 Inhibitors
PSV	Peak Systolic Velocity
RI	Resistive Index
RP	Raynaud’s Phenomenon
SBP	Systolic Blood Pressure
Scl-70	Anti-Topoisomerase I Antibodies
SSc	Systemic Sclerosis
TG	Triglycerides
Tyg	Triglyceride–Glucose Index
VEDOSS	Very Early Diagnosis of Systemic Sclerosis
