# Supplementary material for: Macrovascular Involvement in Systemic Sclerosis: Association Between Carotid Ultrasound Hemodynamics Parameters and Digital Ulcers
Source: Clin Pract. 2025 Aug 18;15(8):152. doi: 10.3390/clinpract15080152 (PMC12384628; doi:10.3390/clinpract15080152)
Supplement: Supplementary file 1 [file clinpract-15-00152-s001.zip › clinpract-3767966-supplementary.pdf]

Supplementary Table S1. Medication usage.

	DU-	DU+	**p-value**
Corticosteroid usage, n/%	7 / 9.2	2 / 22.6	**0.063**
Iloprost current infusion, n/%	66 / 68.8	30 / 96.8	**0.284**
Iloprost duration > 10 years, n/%	39 / 58.2	15 / 51.7	**0.556**
Low dose aspirin, n/%	44 / 57.9	21 / 67.7	**0.344**
Calcium Channel Blockers, n/%	23 / 30.3	11 / 35.5	**0.599**
Sildenafil, n/%	1 / 1.3	6 / 19.4	**<0.001**
Current ERAs, n/%	1 / 1.3	17 / 54.8	**<0.001**
Selexipag, n/%	1 / 1.3	0	**0.521**
Immunosupppresants, n/%	19 / 25	17 / 54.8	**0.003**
Angitensin Receptor Blockers, n/%	5 / 6.6	5 / 16.1	**0.124**
ACE-I, n/%	10 / 13.2	6 / 19.4	**0.415**
Beta-blockers, n/%	11 / 14.5	2 / 6.5	**0.249**
Diuretics, n/%	3 / 3.9	1 / 3.2	**0.858**
Lipids lowering therapy, n/%	13 / 17.1	8 / 25.8	**0.304**
Uric acid lowering therapy, n/%	2 / 2.6	3 / 9.7	**0.117**
